# Characterization of Brca2-Deficient Plants Excludes the Role of NHEJ and SSA in the Meiotic Chromosomal Defect Phenotype

**DOI:** 10.1371/journal.pone.0026696

**Published:** 2011-10-21

**Authors:** Marilyn Dumont, Sophie Massot, Marie-Pascale Doutriaux, Ariane Gratias

**Affiliations:** Institut de Biologie des Plantes, CNRS UMR8618, Université Paris Sud-11, Orsay, France; Universita' di Milano, Italy

## Abstract

In somatic cells, three major pathways are involved in the repair of DNA double-strand breaks (DBS): Non-Homologous End Joining (NHEJ), Single-Strand Annealing (SSA) and Homologous Recombination (HR). In somatic and meiotic HR, DNA DSB are 5′ to 3′ resected, producing long 3′ single-stranded DNA extensions. Brca2 is essential to load the Rad51 recombinase onto these 3′ overhangs. The resulting nucleofilament can thus invade a homologous DNA sequence to copy and restore the original genetic information. In *Arabidopsis*, the inactivation of Brca2 specifically during meiosis by an RNAi approach results in aberrant chromosome aggregates, chromosomal fragmentation and missegregation leading to a sterility phenotype. We had previously suggested that such chromosomal behaviour could be due to NHEJ. In this study, we show that knock-out plants affected in both *BRCA2* genes show the same meiotic phenotype as the RNAi-inactivated plants. Moreover, it is demonstrated that during meiosis, neither NHEJ nor SSA compensate for HR deficiency in *BRCA2*-inactivated plants. The role of the plant-specific DNA Ligase6 is also excluded. The possible mechanism(s) involved in the formation of these aberrant chromosomal bridges in the absence of HR during meiosis are discussed.

## Introduction

One of the most cytotoxic DNA damage is chromosomal breakage, where a DNA double-strand break (DSB) occurs in the duplex DNA. Failure to repair correctly even one DNA DSB can result in the loss of genetic information, chromosome rearrangement, mutations and lead eventually to cell death. In plants, as in other organisms, cells have developed powerful and rapid cellular responses, leading to cell cycle arrest and DNA DSB repair. In eukaryotes, DNA broken ends can be processed by three major DSB repair pathways that are tightly regulated, depending on cell type and cell cycle phase: Non-Homologous End Joining (NHEJ), Single-strand annealing (SSA), and Homologous Recombination (HR).

In the NHEJ pathway, DNA broken ends are simply joined with little or no further processing. In mammalian cells, the Ku70-Ku80 heterodimer forms a ternary complex with the DNA-PKcs, and binds to the DSB. The binding of this complex prevents excessive degradation and promotes the recruitment of other factors involved in the processing of DNA ends to make them suitable for the ultimate step of ligation by the LigaseIV-Xrcc4 complex [1], [2]. While no ortholog of DNA-PKcs has been found in *Arabidopsis*, At*Ku70*, At*Ku80*, At*LigIV* and At*Xrcc4* homologs have been identified [3], [4], [5], [6]. Mammalian null mutants affected in the NHEJ pathway present various orders of phenotype severity. For instance, *ku* mutants are immunodeficient and exhibit an accelerated senescence (in correlation with the deregulation of telomere length), while LigaseIV deficiency leads to embryonic lethality in mice. In *Arabidopsis*, all characterized *nhej* mutants are viable but hypersensitive to various DNA damaging agents, except UV [5]. The *ku* mutants are hypersensitive to menadione (which causes oxidative damage), ionising radiations (X- and gamma-rays) and bleomycin (a radiomimetic), methylmethanesulfonate (MMS, an alkylating agent causing abasic sites and single-strand nicks) [4], [5], [7], [8], [9], [10]. Hypersensitivity to MMS and gamma-irradiation has also been described for *ligIV* mutants [5], [11]. Direct evidence for their involvement in NHEJ comes from plasmid rejoining assays. In protoplasts derived from *ku80* and *ku70* mutant plants, the religation efficiency of plasmids linearized by enzymes generating blunt or 5′overhang ends was significantly reduced [9], [10].

The SSA and the HR pathways are homology-dependent processes for repairing DNA DSB. Both are initiated by the 5′ to 3′ resection of the broken DNA ends in order to uncover extensive single-stranded DNA (ssDNA) 3′ overhangs, a critical intermediate in both SSA and HR. These 3′ ssDNA tails are coated by the single-stranded DNA binding protein, RPA.

After DNA resection, the central step of SSA consists of the annealing between complementary single-stranded DNA sequences on either side of the DSB in a RAD52- and RAD59-dependent, but RAD51-independent, manner. Unpaired non-homologous 3′ tails are then cleaved by the Rad1-Rad10 complex (XPF-ERCC1 in mammals), which is also involved in DNA excision repair, in order to complete the DSB repair with DNA synthesis from the newly cleaved ends and their final ligation. In *Arabidopsis*, mutants affected in At*RAD1* (or *UVH1*) or At*RAD10 (*also called *AtERCC1*) activities have been identified as gamma- and UV-hypersensitive [12], [13], [14]. In contrast to XPF- or ERCC1-deficient mice, the corresponding single mutant plants are viable in the absence of exogenous DNA damaging agents, grow normally and are fertile. Using a plasmid recombination assay, it was shown that each gene was required for the removal of 3′-ended non-homologous DNA single-stranded tails from SSA intermediates, generated by annealing between direct repeats [15], [16], [17], [18], [19].

In contrast to NHEJ and SSA that are inherently error prone, HR is conservative, as it proceeds *via* the copy of the missing sequence from a homologous template. Moreover, HR is required during meiosis for correct chromosome segregation and the generation of genetic diversity. Meiotic recombination is initiated by the introduction of programmed DNA DSB catalyzed by the topoisomerase-like transesterase activity of dimeric Spo11. This leads to a covalent link between the catalytic tyrosine of a Spo11 monomer and the 5′ DNA end on both sides of the DSB. In budding and fission yeast, removal of each Spo11 occurs by endonucleolytic cleavage several nucleotides downstream from the 5′ end, catalyzed by the Mre11-Rad50-Xrs2 complex and Sae2. This releases a Spo11 monomer bound to an oligonucleotide, sometimes called a “spolligo” [20], [21], [22], [23].

The repair process of HR in somatic and meiotic cells is initiated by extensive processing of DNA ends, uncovering 3′ ssDNA stretches that become coated by RPA. This resection is essential for the establishment of a recombinase-DNA nucleofilament on the 3′ single strand, which performs the homology search for a target DNA sequence to use as a template to copy, either the sister chromatid in somatic cells or a homologous chromosome in meiotic cells. Two recombinases can be loaded onto the ssDNA extension to mediate the strand displacement and homology search: the ubiquitous Rad51, the eukaryotic RecA homolog, and its homolog Dmc1 that has a specific role during meiosis. Once a homology is found, DSB repair is completed by DNA synthesis using the homologous sequence as a template and religation follows [24].

The displacement of RPA and its replacement by the recombinases rely on mediator proteins, such as the Rad51 paralogs, Rad52 and/or Brca2, which exist in most eukaryotes. In humans, *BRCA2* gene mutations are associated with hereditary breast cancer [25], [26] and genome instability [27], [28]. In mice, the knockout of *BRCA2* leads to early embryonic lethality associated with chromosomal rearrangements [29]. Structural and biochemical studies have shown the interaction between Rad51 and Brca2 [30], [31], [32]. Together with their co-localization in nuclear foci, after DNA damaging treatment of the cells, this definitively links Brca2 to homologous recombination [33].

Recently, the human Brca2 protein was purified [34], [35], [36]. It appears that one Brca2 molecule binds approximately six Rad51 monomers and that Brca2 stimulates the binding of Rad51 onto ssDNA even when it is covered by RPA. This interaction is mediated through the specific BRC domains which are present in all Brca2 proteins, but in varying numbers depending on species. For example, eight BRC domains are found in human Brca2 [31], whereas only one is present in Brh2 and Ce-BRC2, the Brca2 homologs of *Ustilago maydis* and *Caenorhabditis elegans* (*C. elegans*), respectively [37], [38]. In *Arabidopsis*, two At*BRCA2* genes have been identified: on chromosomes IV (At*BRCA2(IV),* also named At*BRCA2a*) and V (At*BRCA2(V)* or At*BRCA2b*). They encode two proteins of 1511 (At*BRCA2a*) and 1155 amino acids (At*BRCA2b*), which share 94.5% identity and contain four BRC motifs each. The two *Arabidopsis* genes are expressed in floral buds and the proteins they encode have been shown to interact with both Rad51 and Dmc1, the meiotic-specific recombinase [39], [40]. Recently, the Brca2-Dmc1 interaction has been confirmed in humans [41]. These data thus linked Brca2 to meiotic recombination for the first time.

The understanding of Brca2 function has been considerably hampered by the early embryonic lethality associated to knocking out BRCA2 in mouse. Clear evidence for the meiotic role of Brca2 came from *A. thaliana* and *C. elegans* since the absence of the Brca2 function is viable and only leads to sterility due to meiotic defects in both models [38], [39]. Indeed, RNAi-inactivation of both *Arabidopsis BRCA2* genes, specifically during meiosis, caused sterile plants resulting from an improper meiosis with chromosomal aberrations: absence of bivalent formation, chromosomal entangling, bridges and fragmentation. This phenotype was dependent on the formation of meiotic DNA double-strand breaks as it was alleviated in a *spo11* mutant [39]. We hypothesized that in *A. thaliana* the chromosomal abnormalities observed upon depletion of Brca2 at meiosis could be the result of an alternative repair of the meiotic DSB, in the absence of HR [39]. In *C. elegans*, Martin et al. (2005) showed that the RNAi depletion of *LIGIV* significantly reduced meiotic chromosome aggregation in *Cebrc-2* single mutants and could give rise to chromosomal fragmentation. These observations suggested that NHEJ could be partially responsible for the aberrant chromosome fusions in the absence of Ce*BRC-2*.

In this study, the meiotic defects previously observed in Brca2-inactivated plants were confirmed in *brca2* double mutant plants containing a T-DNA insertion in each At*BRCA2* gene. The potential role of alternative DNA repair pathways in the meiotic phenotype was tested by inactivating Brca2 in *nhej* and/or *ssa* mutant backgrounds. We demonstrate that neither NHEJ nor SSA were responsible for the observed cytological defects. Moreover, based on the hypothesis that covalent repair is responsible for the observed meiotic chromosomal defects in the absence of Brca2, we tested the role of a recently characterized plant-specific DNA ligase, AtLigase6. Since the abnormal meiotic figures were maintained in *lig6* plants inactivated for Brca2, the role of this DNA ligase during meiosis in the absence of HR was excluded.

## Results

### A *brca2* double mutant exhibits the same meiotic phenotype as Brca2-inactivated plants

**Figure 1:**
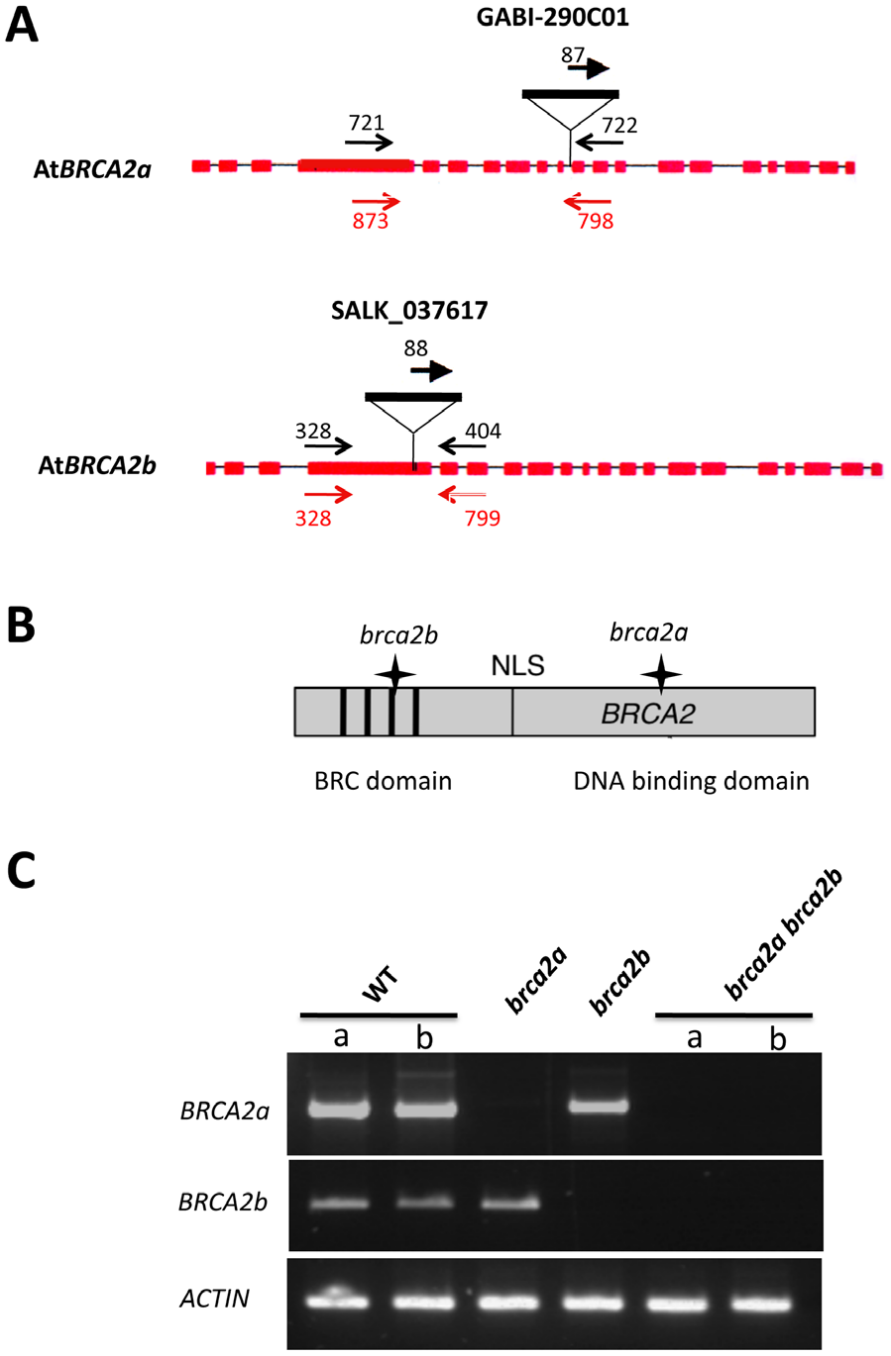
The *brca2* single and double mutants. **(A)** Position of the T-DNA insertions in At*BRCA2a* and At*BRCA2b*. The structure of the At*BRCA2*a and *AtBRCA2b* genes is represented by shaded boxes (exons) and thin lines (introns). The T-DNA insertion position is indicated. Each primer pair used to identify the mutants by PCR are compiled on the diagram in black and primer pairs used for RT-PCR analyses are given in red; their localization is correct but not to scale. **(B)** Schematically represented Brca2 protein with the position of the BRC repeats and the NLS relative to the T-DNA insertions, as indicated by a star. For convenience, and because they share 94.5% of identity, a single Brca2 protein is represented. **(C)** RT-PCR analysis of At*BRCA2* transcripts in the single and double *brca2* mutants. RNA was extracted from young floral buds of wild-type plants (2 different plants, a and b) as well as of *brca2a*, *brca2b* and *brca2a brca2b* (2 different plants, a and b) mutant plants and was then reverse-transcribed. Double-stranded cDNAs were then PCR-amplified using the primer pairs represented in red in Figure 1A. The constitutive *ACTIN* gene transcript was used as a control.

**Figure 2:**
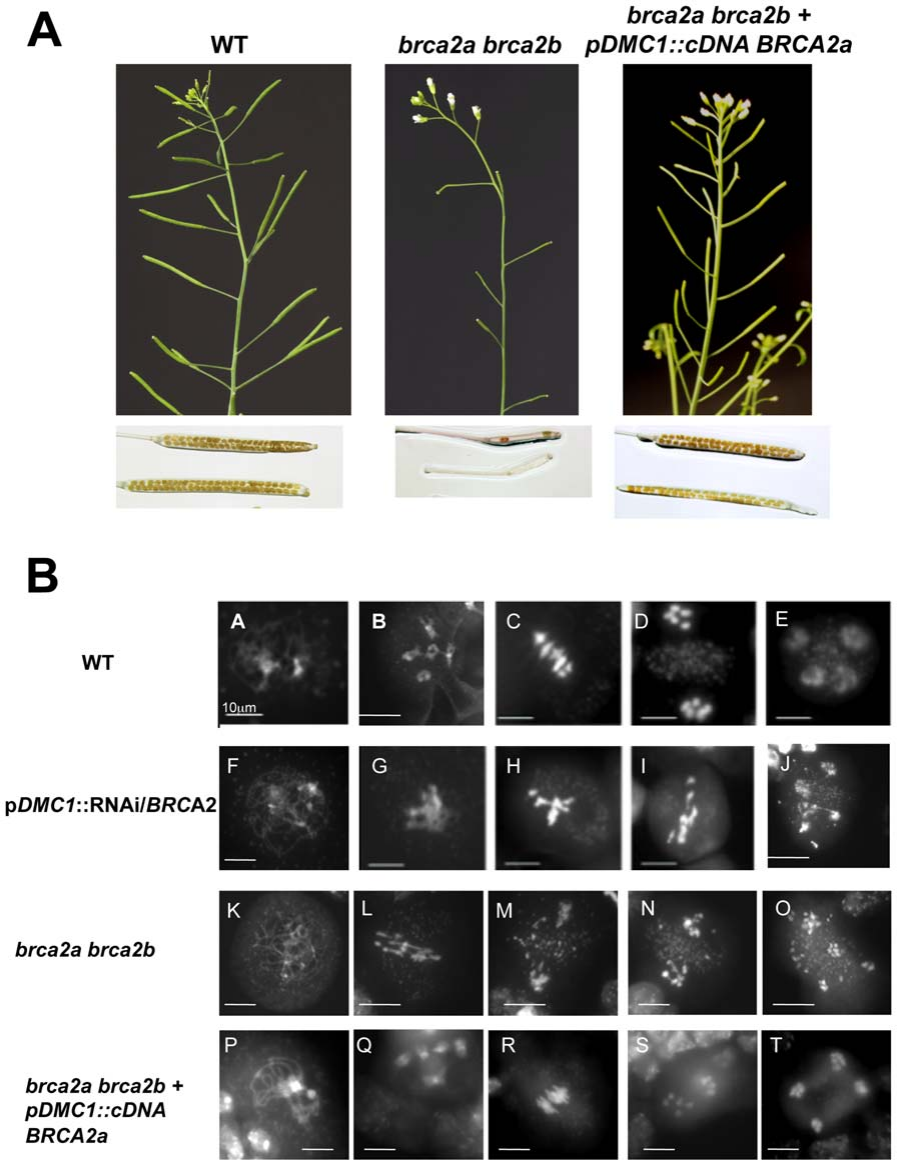
Meiotic defects in *brca2a brca2b* mutant plants and in wild-type Brca2-inactivated plants. **(A)** Wild-type and *brca2* double mutant plants exhibt no growth defect except for sterility. Chloralhydrate discolored siliques are full of seeds in wild-type plants in comparison with the discolored siliques of the *brca2* double mutant plants. **(B)** Observation of meiocytes by DAPI staining in Brca2-deficient plants, transformed or not with the full length cDNA of At*BRCA2a*, and in *brca2a brca2b* homozygous double mutant plants. (A–E) Different stages of meiosis in the wild-type plants. Meiosis is normal. (A) Prophase I stage, (B) diakinesis, the five bivalents are attached by a chiasma, (C) metaphase I with five aligned bivalents, (D) anaphase I, bivalents segregate into two sets of five univalents, (E) anaphase II, with four groups that contain five chromosomes each after sister chromatid separation. (F–J) Different stages of meiosis in wild-type plants transformed with the p*DMC1*::RNAi/*BRCA2* construct. (F) Prophase I, (G) no normal diakinesis phase (H) metaphase I with condensed and entangled chromosomes, (I) anaphase I, with entangled and stretched chromosomes. (J) Anaphase II, with bridges extending between chromosomes. (K–O) Different stages of meiosis in *brca2* double mutant plants. (K) Prophase I, (L) anaphase I, entangled and stretched chromosomes. (M) Metaphase II with entangled chromosomes. (N) anaphase II, fragmentated chromosomes. (O) telophase II with chromosome missegregation. (P–T) Different stages of meiosis in *brca2* double mutant plants, transformed with the p*DMC*1::cDNA At*BRCA2a*. Meiosis is restored to normal. (P) Prophase I stage, (Q) diakinesis, (R) metaphase I, (S) anaphase I, (T) anaphase II. Bar 10 µm.

In a previous study, At*BRCA2a* and At*BRCA2b* expression was inactivated during meiosis by RNAi using an inverted 510 pb-fragment of the *BRCA2* cDNA under the control of the meiotic-specific promoter of *DMC1* (p*DMC1*) [39]. In this work, single and double T-DNA insertion mutants for *AtBRCA2* were isolated and their phenotype compared to the RNAi-inactivated plants (named p*DMC1*::RNAi/*BRCA2* ). First, *brca2* plants mutated in the At*BRCA2* genes *via* either a T-DNA insertion located in the 10^th^ intron of At*BRCA2a* (in the Cter DNA binding domain) or an insertion in the 4^th^ exon of At*BRCA2b* (in the Nter domain of the protein, containing the BRC motifs) were isolated (Figure 1A and Figure 1B). At*BRCA2* transcripts were analysed by RT-PCR, using primers flanking the insertion sites in wild-type and in *brca2* single mutant plants. Transcripts of the disrupted genes were not detected in the corresponding mutant lines, whereas transcripts of each At*BRCA2* gene were amplified in wild-type plants. This strongly suggested that the two single *brca2* lines were null mutants (Figure 1C). Each single mutant showed normal development and fertility. By crossing the single mutants, the double *brca2a brca2b* mutant was obtained. These latter plants showed no growth defect and behaved as the wild-type under normal greenhouse conditions. However, they were partially sterile producing very short and mostly empty siliques (Figure 2A). Moreover, the presence of meiotic defects was observed after DAPI staining of the chromosomes in the meiocytes. Indeed, all meiotic figures showed chromosomal entangling without bivalent formation, bridges and fragmentation, leading to chromosomal missegregation (Figure 2B) as previously described for p*DMC1*::RNAi/*BRCA2* plants. A transgene containing a full length At*BRCA2a* cDNA under the control of the promoter of the meiotic recombinase Dmc1 (p*DMC1*::cDNA At*BRCA2a*) was introduced in 13 *brac2a brca2b* double mutant plants. 11 transformant plants presented a restored phenotype: 9 were completely fertile as demonstrated by the observation of wild-type siliques content and normal meiosis (Figure 2) and 2 were partially fertile (as they presented some siliques that developed as sterile). Only 1 transformant was sterile with developmental defects. As a control, 11 *brca2a brca2b* double mutant plants were transformed with a transgene containing the p*DMC1*::RNAi/0 construct, corresponding to the “empty vector” [39]: all of them were sterile (data not shown). These results reinforce the evidence for the role of At*BRCA2* at meiosis, previously uncovered by our RNAi strategy.

### Characterization of *nhej* and *ssa* mutant plants by RT-PCR and under various genotoxic stress

**Figure 3:**
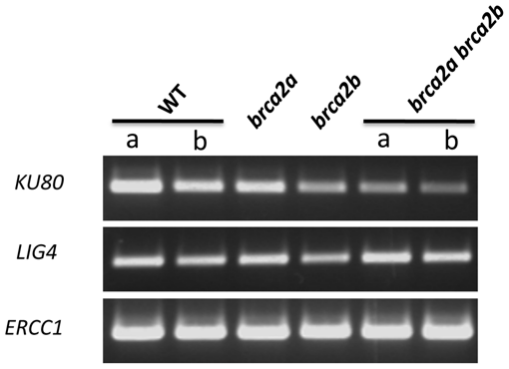
RT-PCR analysis of genes involved in NHEJ and SSA in the single and double *brca2* mutants. RNA was extracted from young floral buds and reverse-transcribed, as described in Figure 1C. Double-stranded cDNAs were PCR-amplified using primer pair 454/455 for At*KU80* (see primer positions in Figure 4 and sequences in Table1), 336/445 for At*LIGIV* and 452/453 for At*ERCC1* (see Table 1 for sequences). The constitutive *ACTIN* gene transcript used as a control is presented in Figure 1C.

**Figure 4:**
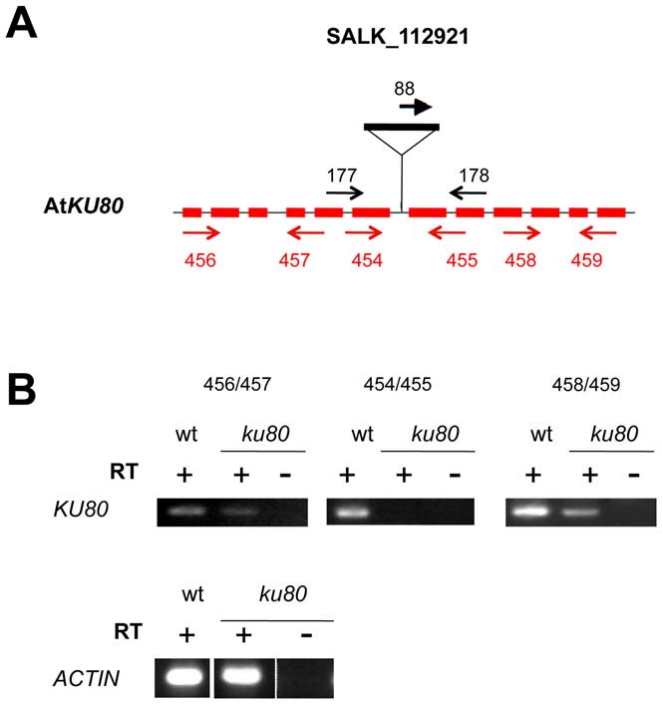
T-DNA insertion and expression in *ku80* mutant. **(A)** Position of the T-DNA insertion in At*KU80*. The structure of the At*KU80* gene is represented by shaded boxes (exons) and thin lines (introns). The T-DNA insertion position is indicated. Each primer pair used to characterize the mutant by PCR are indicated in black and primer pairs used for RT-PCR analyses are given in red; their localization is correct but not to scale. **(B)** RT-PCR analysis of At*KU80* transcripts in *ku80*-/- mutant plants. RNA, extracted from floral buds of wild-type or *ku* mutant plants was reverse-transcribed. Double-stranded cDNAs were amplified by RT-PCR, performed with three different primer pairs: 5′ or 3′ to the T-DNA and flanking the T-DNA insertion. For primer positions, see above (Figure 4A). The constitutive *ACTIN* gene was used as a control.

In order to identify the molecular pathways involved in the aberrant cytological phenotype observed in the Brca2-deficient plants during meiosis, mutant plants deficient in either the NHEJ (*ku80^-/-^* and *ligIV^-/-^*) or the SSA (*ercc1^-/-^*) pathways were characterized. Examining amplification of these transcripts specifically in meiocytes was not possible, as meiocytes would have to be specifically dissected which is technically difficult. However, as shown in Figure 3, all these three genes, and thus the pathways they are involved in, were found expressed in young flower buds, where meiosis takes place, in single as well as in double *brca2* mutant plants. Two mutant lines have been previously described: SALK_044027, where the T-DNA insertion is in exon 6 of the At*LIGIV* gene [42], [43] and SALK_033397 which contains a T-DNA insertion in exon 3 of At*ERCC1* [16]. The absence of transcripts corresponding to the affected gene was confirmed for each mutant line by RT-PCR using primers flanking each T-DNA insertion (data not shown). The *ku* mutant line used in this study (SALK_112921) had not been characterized to date. It contains a T-DNA in the 6^th^ intron of the At*KU80* gene (Figure 4A). RT-PCR analysis of the 5′ and 3′ regions flanking the T-DNA insertion revealed the presence of At*KU80* transcripts in both wild-type and *ku80* mutant plants (Figure 4B). However, no transcripts could be detected in *ku80* mutant plants when primers flanking the T-DNA insertion were used, suggesting that splicing of the 6^th^ intron did not occur in the *ku80* mutant. As the insertion site is positioned in the region encoding the domain involved in hetero-dimerization with Ku70, it is most likely that a putative protein, lacking this domain, would be non-functional. Thus, these *ku80* plants were considered as functional null mutants. The mutant plants, whatever the affected DNA repair pathway, exhibited no obvious developmental defects under normal growth conditions and were fertile, as previously described for *ercc1*, *ku80* and *ligIV Arabidopsis* mutants [9], [13], [16].

We believed that in the absence of HR during meiosis, the different DNA DSB repair pathways could compensate for each other. Thus, *nhej* mutant plants, *ku80* and *ligIV,* were crossed with *ssa* mutant plants, *ercc1,* and double *ku80 ercc1* and *ligIV ercc1* mutants affected in both pathways were isolated and genotyped. Both double mutants were viable, presented no obvious developmental defects under normal growth conditions and were fertile.

**Figure 5:**
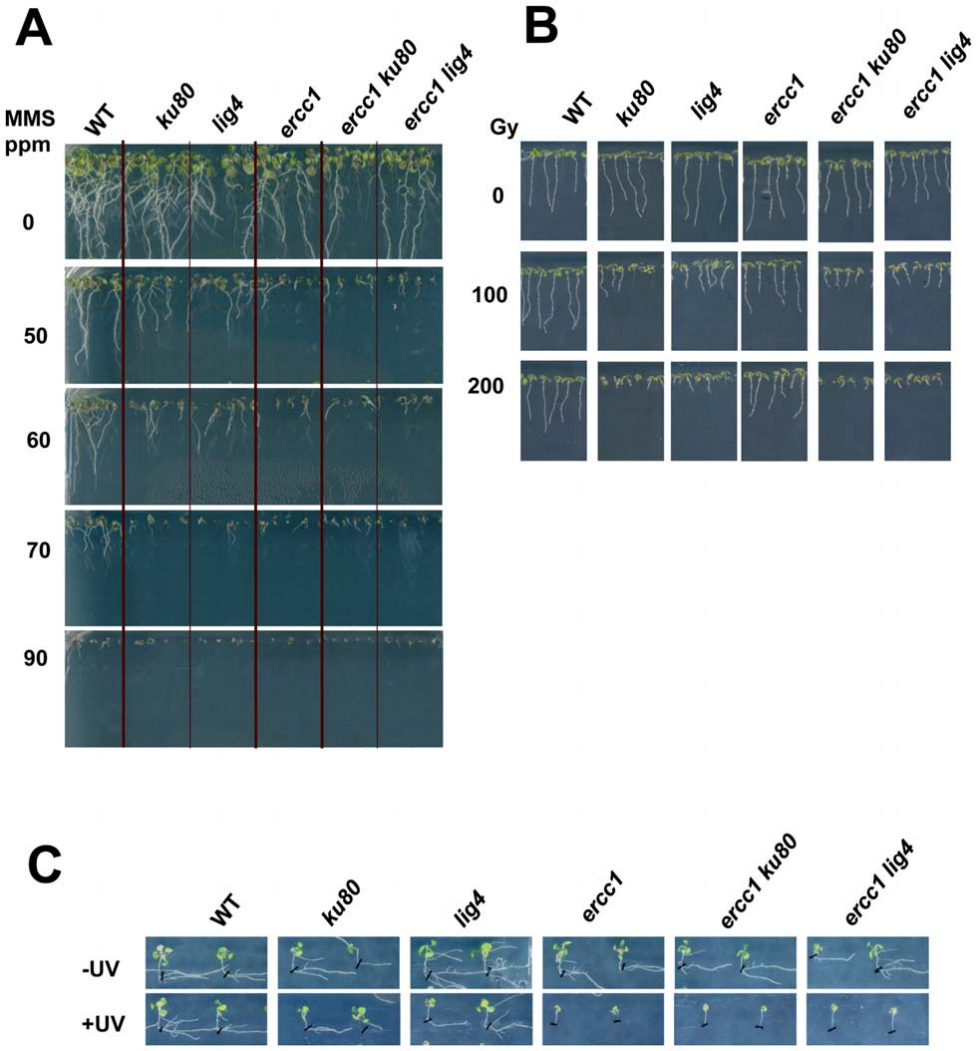
Hypersentivity to MMS, gamma-rays and UV irradiation of *nhej*, *ssa* and *nhej ssa* plants. Before sowing, all seeds were surface-sterilized. **(A)** MMS hypersensitivity, 11 days post-germination. Seeds were sown on MS 0.5 agar 1% sucrose supplemented with MMS at various doses. **(B)** Gamma-irradiation hypersentivity, 7 days post-irradiation. After 48 h at 4°C in darkness, seeds were exposed to various doses of gamma-rays : 0, 100 and 200 grays before being sown on MS 0.5 agar. **(C)** UV hypersensitivity, 10 days post-irradiation. Seeds were sown in MS 0.5 agar. After 4 days of growth, the plantlets were exposed to UV-C, left in the dark for 3 days to avoid photoreactivation, and then exposed to light.

Sensitivity to various DNA damaging agents is a classical assay to characterize DNA repair mutant plants as most of them show no obvious somatic phenotype. To control that our mutants were indeed affected in DNA repair, their sensitivity to MMS, gamma-ray and UV irradiation was assayed. In comparison to wild-type plants, root growth was affected in the *nhej* plants as well as in the *ssa* plants in the presence of MMS or after gamma exposure. Indeed, the MMS hypersensitivity was visible at 50 ppm and gamma–ray hypersensitivity was observed at 100 grays for each single mutant line. However, MMS-induced retarded growth was more pronounced in *ercc1* than in *ku80* and *ligIV* plants (Figure 5A). MMS is a methylating agent, and due to the occurrence and clustering of modified bases, it can generate both SSB and DSB, which is reflected in the fact that *ercc1* mutants (deficient for both SSA and BER) appeared to be more sensitive to this genotoxic treatment. Reciprocally, *ercc1* plants were less sensitive to gamma irradiation when compared to *ku80* and *ligIV* (Figure 5B). Ionising radiations mainly give rise to clustered DNA damages (modified bases and abasic sites) that lead to DNA DSB. Such DNA strand breaks are mostly repaired by NHEJ as suggested by the higher hypersensitivity of *ku80* and *ligIV* mutants to gamma-rays. Finally, as expected, only the *ercc1* plants were hypersensitive to UV exposure (Figure 5C). All of these results confirmed that the different mutant *Arabidopsis* lines were affected in DNA DSB repair.

MMS and gamma-ray sensitivity of the double *nhej ssa* mutants were assessed in comparison to the single mutant plants (Figure 5). For each stress, we noted that the sensitivity of the double mutant was similar to that observed for the most affected single mutant: *ku80 ercc1* and *ligIV ercc1* appeared to be hypersensitive to MMS and UV as was the *ercc1* single mutant, whereas they showed a similar hypersensitivity to gamma-rays as the *nhej* single mutant. Therefore, no cumulative effect was observed.

### The *brca2* meiotic phenotype is maintained during meiosis in *nhej* and *ssa* backgrounds

The Brca2 function was inactivated in the *nhej* and *ssa* mutant plants by transforming the mutant plants with the previously used p*DMC1*::RNAi/*BRCA2* construct. As a control, mutant plants were also transformed with a p*DMC1*::RNAi/0 construct containing no insert [39]. No somatic phenotype was observed in any of the transformed plants containing the “empty” construct or the p*DMC1*::RNAi/*BRCA2* construct. When flowers emerged, all plants containing the control construct were fertile, whereas most of the mutant plants transformed with p*DMC1*::RNAi/*BRCA2* were partially sterile in the single *nhej* or *ssa* mutants (between 67 to 80%) as well as in the double *nhej ssa* mutants (between 60 to 78%), as previously observed for wild-type p*DMC1*::RNAi/*BRCA2* transformed plants.

**Figure 6:**
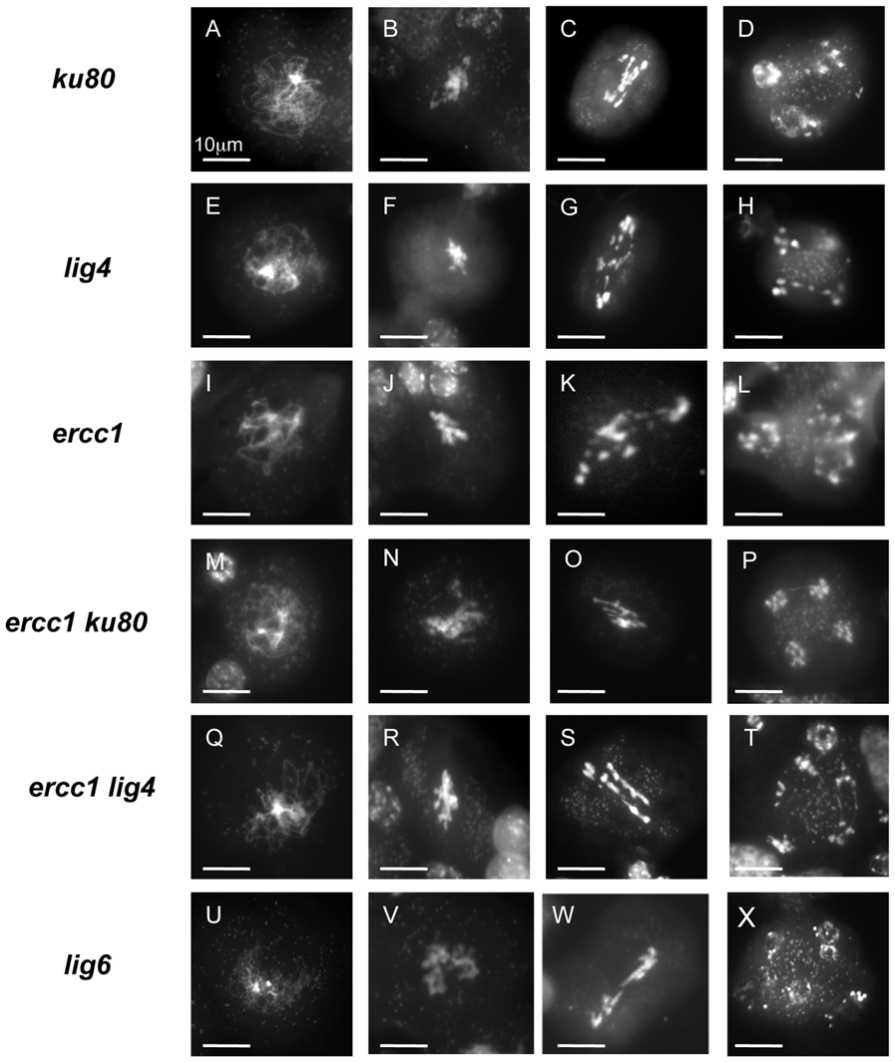
Observation of meiocytes by DAPI staining in *nhej*, *ssa*, *nhej ssa* and *lig6* mutant plants transformed with the p*DMC1*::RNAi/*BRCA2* construct. Different stages of meiosis were observed in plants transformed with p*DMC*1::RNAi/*BRCA2* in *nhej* mutant plants, *ku80* (A–D) or *lig4* (E–H), and in *ssa* mutant plants, *ercc1* (I–L), in *nhej ssa* double mutant plants, *ercc1 ku80* (M–P) or *ercc1 lig4* (Q–T) and in *lig6* mutant plants (U–X). (A, E, I, M, Q, U) prophase I. (B, F, J, N, R, V) metaphase I. (C, G, K, O, S, W) anaphase I. (D, H, L, P, T, X) anaphase II. Bar 10 µm.

The meiotic behaviour was examined after DAPI staining of the chromosomes in the meiocytes of several independent transformed plants that were inactivated for the Brca2 function: 175 meiotic figures from two *ku80*, 170 meioses from two *ligIV* and 34 meioses from two *ercc1* lines independently transformed with the p*DMC1*::RNAi/*BRCA2* construct were observed. As a control, they were compared to the meioses of one *ku80*, one *ligIV* and one *ercc1* plant containing the RNAi/0 construct. All of the observed control plant meiotic figures were normal in the single mutants affected for either NHEJ *(ku80, ligIV*) or SSA (*ercc1*), as well as in the *nhej ssa* double mutant (Supplementary Figure S1). On the other hand, meiosis was profoundly disturbed in meiocytes of these same mutant lines transformed with the p*DMC1*::RNAi/*BRCA2* construct: chromosomal entangling without bivalent formation, fragmentation, and missegregation of chromosomes (Figure 6). Such observations have been previously reported in wild-type p*DMC1*::RNAi/*BRCA2* plants [39]. These observations suggested that, contrary to our hypothesis, in the absence of Brca2 during meiosis, neither NHEJ nor SSA were responsible for an alternative meiotic DSB repair that would have been revealed because of the absence of HR [39]. The impact of the inactivation of both pathways in the absence of Brca2 during meiosis was also examined. Meiotic figures from one p*DMC1*::RNAi/*BRCA2* transformant for *ercc1 ku80* (257 meiotic events, among them 80 were post-prophase) and two p*DMC1*::RNAi/*BRCA2* transformants for *ercc1 lig4* (115 meiosis, including 63 post-prophase stages) were observed. In all double mutant plants transformed with p*DMC1*::RNAi/*BRCA2*, the *brca2* meiotic phenotype remained unaltered (Figure 6).

All of these results suggest that 1) the aberrant chromosomal figures observed in the absence of Brca2 during meiosis are not due to NHEJ or SSA and 2) the other major DNA DSB repair pathways, in the absence of HR, do not compensate for each other during meiosis.

### DNA Ligases in *Arabidopsis*

Our initial hypothesis was that the chromosomal bridges detected in the “failed” anaphases in the absence of Brca2 were due to covalent DNA links, probably between non-homologous chromosomes. Since our data exclude the role of NHEJ and SSA, all DNA ligases apart from LigaseIV (the NHEJ specific enzyme already studied in this work) could be potentially incriminated. The *Arabidopsis* genome contains three other sequences encoding DNA ligases: At*Ligase1* which is involved in replication and Base Excision Repair (BER), At*Ligase1a* which shares 71% identity with At*Ligase1* but for which no transcripts could be detected (our personal data and transcriptome analyses: http://csbdb.mpimp-golm.mpg.de/csbdb/dbxp/ath/ath_xpmgq.html), suggesting that it may be a pseudogene, and At*Ligase6*, a plant specific ligase that appears to be involved in seed longevity [44]. AtLigase6 has a highly conserved DNA ligase catalytic domain and a beta-lactamase domain containing a beta-CASP motif found in Artemis and other proteins known to play a role in nucleic acid processing [45], [46]}. Since *lig1* mutant plants are embryonic lethal [47], [48], we thus examined whether the plant specific AtLigase6 could be involved in the meiotic phenotype of the Brca2-deficient plants.

**Figure 7:**
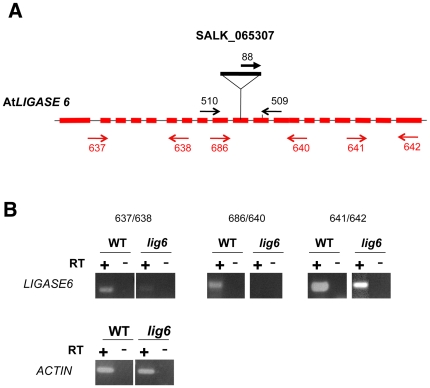
T-DNA insertion and expression in *lig6* mutant. **(A)** Position of the T-DNA insertion in At*LIG6*. The structure of the AtLIG6 gene is represented by shaded boxes (exons) and thin lines (introns). The T-DNA insertion position is indicated. Each primer pair used to identify the mutants by PCR are indicated in black while primer pairs used for RT-PCR analyses are given in red; their localization is correct but not to of scale. **(B)** RT-PCR analysis of At*LIG6* transcripts in *lig6*-/- mutant plants. RNA, extracted from floral buds of wild-type or *lig6* mutant plants was reverse-transcribed. Double-stranded cDNAs were amplified by RT-PCR, performed with three different primer pairs: 5′ or 3′ to the T-DNAand flanking the T-DNA. The position of each primer is given above (Figure 7A). The constitutive *ACTIN* gene was used as a control.

Homozygous *lig6* plants containing a T-DNA insertion in exon 11 of the gene were obtained from the SALK collection (SALK_065307) (Figure 7A). All plants grew normally, they were fertile and undertook normal meioses (data not shown). These observations are in agreement with what was previously observed in a different *lig6* insertional line (Waterworth et al, 2010) [44]. RT-PCR analyses detected transcripts on both sides of the T-DNA insertion but no transcripts could be found when primers flanking the T-DNA insertion were used (Figure 7B). As the T-DNA insertion is positioned in an exon, 42 bp from the codon of the catalytic lysine just upstream from the conserved motif II [49] lying in the core domain, and more specifically around the nucleotide binding pocket responsible for the nucleotidyl transfer, the catalytic activity of a putatively expressed protein in this mutant is probably non-functional.

### DNA Ligase6 is not responsible for the *brca2* meiotic phenotype

Waterworth et al. (2010) observed a slight but significant growth hypersensitivity of *lig6* plants after a 100 gy X-ray irradiation, leading them to suggest that At*Ligase6* could play a minor role in the repair of X-ray induced DNA damage. Transformation of our *lig6* mutant plants with p*DMC1*::RNAi/*BRCA2* was performed to inactivate the Brca2 function in *lig6* plants. 87% of the transformants (26/30) were partially sterile while *lig6* plants or p*DMC1*::RNAi/0 transformed plants (six transformed plants, 57 meioses observed from two independent transformants) were normally fertile (Supplementary Figure S1). After DAPI staining of the chromosomes in the meiocytes of seven *lig6* plants, independently transformed with the p*DMC1*::RNAi/*BRCA2* construct (426 meiotic figures, including 219 post-prophase events), the b*rca2* meiotic phenotype was consistently observed (Figure 6), thus excluding a role of At*Ligase6* in this phenotype.

## Discussion

In *Arabidopsis*, RNAi-inactivation of Brca2 during meiosis gave rise to a sterility phenotype due to an aberrant meiosis characterized by an absence of bivalent formation, chromosomal entangling, fragmentation and missegregation. Such defects were Spo11-dependant, therefore an alternative DNA repair process was proposed to be responsible for an aberrant repair of meiotic DSB in the absence of HR. In this study, we show that *brca2* double mutant plants exhibit a similar meiotic phenotype when compared to the p*DMC1*::RNAi/*BRCA2* transformed plants. Moreover, our data clearly exclude the role of NHEJ and SSA in the aberrant meiotic chromosomal figures of Brca2-deficient plants.

### Phenotypic characteristics of Brca2-deficient plants

In this study, double *brca2a brca2b* mutant plants were shown to have no obvious phenotype in terms of vegetative growth, contrary to the occasional fasciation described by Abe et al. (2009). This may be explained by the use of different ecotypes. However, the double *brca2* mutant displayed the same meiotic phenotype as previously described for p*DMC1*::RNAi/*BRCA2* transformed plants. Each single mutant was fertile, indicating the functional redundancy of the two At*BRCA2* genes at meiosis [50], [51]. This could not have been concluded from the p*DMC1*::RNAi/*BRCA2* transformed plants, as both At*BRCA2* genes were silenced by the RNAi construct. Previously it was found that p*DMC1*::RNAi/*BRCA2* transformed plants produced a few seeds that could have arisen from a partial silencing of the At*BRCA2* genes. However, this does not appear to be the reason since in the present study, a few seeds were also produced by the double mutant plants (Figure 2A). Preliminary experiments showed that the seeds germinated, producing *brca2* double mutant plants that developed normally, although not fertile. This could mean that in the absence of Brca2, HR could be partially functional and give rise to some rare normal meiosis events that were not detected in our observations. Alternatively, the abnormal meiosis we observed may not be always detrimental to the chromosomes. It will be of interest to follow the *brca2* cumulative phenotypes from generation to generation, to check if their meiotic (and somatic) phenotypes become exacerbated.

### NHEJ and SSA do not compensate for HR deficiency during meiosis

Our analyses of the chromosomes in the meiocytes by DAPI staining in the double *brca2a brca2b* mutants revealed that the depletion of Brca2 during meiosis led to the absence of bivalent formation and to chromosome aggregates, thus confirming our previous study of plants transformed with the p*DMC1*::RNAi/*BRCA2* construct. In anaphase I, aberrant bridges between chromosomes were systematically observed. We hypothesized that these defects were due to covalent repair of meiotic DBS. NHEJ was the main candidate pathway we believed responsible for these aberrant chromosomal figures. FISH experiments would not have proven that the chromosomes involved in these anaphase bridges were covalently linked, just that they were occasionally aberrantly “associated”. Thus, the Brca2 function was inactivated in NHEJ- but also in SSA-deficient plants. In contrast to *C. elegans*, a role of NHEJ in these meiotic defects can now be excluded, since these aberrant chromosome aggregates were still present in the meiocytes of plants defective in NHEJ [38]. A similar conclusion can be drawn in the case of the SSA-deficient plants. Furthermore, the additive disruption of both the NHEJ and SSA pathways did not modify the *brca2* meiotic phenotype. This demonstrates that, in contrast to somatic cells where deletions and translocations can occur in mutants defective in HR due to the error-prone repair of accidental DNA DSB *via* NHEJ or SSA, neither of these two major DNA DSB repair pathways can compensate for the absence of HR during meiosis in *Arabidopsis*. During meiosis, as the introduction of DNA DSB is programmed, inhibition of the NHEJ and SSA pathways must be very strong to prevent them to compete for or to replace HR. More generally, it is conceivable that the DNA repair processes that are initiated by programmed DNA DSB must be very carefully controlled. If neither NHEJ nor SSA are responsible for the meiotic defects of Brca2 deficient plants, we cannot exclude the role of alternative DNA DSB repair pathways, such as the backup end-joining pathway involving Xrcc1[52]. Hence, further studies should be addressed to analyse meiosis in triple mutants, deficient for all three pathways, in Brca2-inactivated plants. However, recent data suggest that DNA DSB are still repaired in somatic cells of irradiated plants, defective for HR, SSA, NHEJ and backup end-joining, suggesting that other DNA DSB repair process probably remain to be discovered [53].

### Covalent repair or not ?

To better understand the molecular mechanisms responsible for the chromosomal bridges observed during meiotic anaphases in the absence of Brca2, we investigated the putative role of DNA ligases. Four potential DNA ligases have been identified in *Arabidopsis*: the essential At*LIGASE1* involved in DNA replication and BER, At*LIGASE1a*, the NHEJ-specific At*LIGASEIV*, and the plant-specific At*LIGASE6*. As At*LIGASE1a* seems to be a pseudogene, it was excluded from our study. Our results show that the NHEJ specific DNA LigaseIV and the plant-specific Ligase6 were not involved in the meiotic chromosomal defects resulting from the absence of Brca2. Thus, a putative role of a plant DNA ligase activity remains an open question. It is difficult to study the role of DNA Ligase1 as it is essential to DNA replication driving homozygous *lig1* mutant plants to be embryo-lethal [47], [48]. In order to by-pass the lethality of the *lig1* mutant, Waterworth et al (2009) reduced the expression of At*LIGASE1* using an RNAi construct that was set under the control of the ubiquitous CaMV35S promoter [47]. The partially inactivated plants exhibited precocious flowering but as their growth and development were strongly affected, it was difficult to describe them as clearly fertile. Hence, it would be interesting to undertake a meiosis-specific inhibition of At*LIGASE1* expression using the p*DMC1* meiotic promoter, as previously carried out for Brca2. Otherwise, another possibility to consider is that the chromosomes are not covalently linked and that other proteins involved in chromatid cohesion and synapsis could help maintain the aberrant chromosome associations observed in the absence of Brca2.

## Materials and Methods

### Plant material and growth conditions

*Arabidopsis thaliana* Colombia ecotype were used in this study. Mutant lines were identified in the T-DNA express database of the Salk Institute Genomic Analysis Laboratory (http://signal.salk.edu). The insertional mutant affected in At*ERCC1* (line SALK_033397) and in At*LIGIV* (line SALK_044027) have been described previously [16], [42], [43] as well as the At*BRCA2b* insertion line (SALK_ 037617) [50]. The newly characterized mutant lines were GABI_290C01 for At*BRCA2a,* SALK_112921 for At*KU80* and SALK_065307 for At*LIGASE6*. Wild-type and mutant *Arabidopsis* plants were cultivated in a greenhouse at 23°C under long day conditions (16 h light, 8 h dark, humidity 75%).

### Isolation of genomic DNA and genotyping of the plants

Plants were genotyped by PCR performed on genomic DNA extracted from leaves of 2–3 week-old plants in Edwards' buffer [54]. 1/50 of the extracted DNA was used as a template for PCR with two gene-specific primers and one primer specific for the left border of the T-DNA (Salk or Gabi-Kat, depending on the mutant lines) in separate reactions (see Table 1 and Figures 1, 4 and 7). The wild-type allele was amplified with oligonucleotides 721/722 for the At*BRCA2a* locus, 328/404 for At*BRCA2b,* 177/178 for At*KU80,* and 510/509 for At*LIGASE6*. The mutant allele was detected using primer 88 (LBa1) for SALK T-DNA lines or 87 (08409) for Gabi-Kat T-DNA lines and primer 772 for the At*BRCA2a* locus, 404 for At*BRCA2b,* 178 for At*KU80,* or 509 for At*LIGASE6*. PCR reactions were performed in a 20 µl final volume, with 0.2 mM of dNTPs, 5 mM MgCl_2_, 1 µM of each primer, 1U of Taq DNA polymerase (Invitrogen™). They were incubated in a 2720 Thermocycler (Applied Biosystem) at 94°C for 2 min, followed by 35 cycles at 94°C for 30 s, 50°C for 30 s and 72°C for 1 min, except for the PCR on the At*BRCA2* genes where the annealing step was performed at 52°C for 30 s. The PCR samples were then visualized after migration on 0.7% agarose gels in the presence of ethidium bromide.

**Table 1:** Sequence and use of primers in this study.

Name	Gene	DNA sequence (5′-3′)	Use
87	Gabi T-DNA o8409	ATATTGACCATCATACTCATTGC	genotyping
88	Salk T-DNA LBa1	TGGTTCACGTAGTGGGCCATCG	genotyping
721	At*BRCA2a* (At4g00020/10)	GATTGTGCTCTGAATGCTAC	genotyping
722	At*BRCA2a* (At4g00020/10)	CAATTTCTTTACCTTGAGGA	genotyping
873	At*BRCA2a* (At4g00020/10)	ATGAGACCGATTGTGCTCTGAATGC	RT-PCR
798	At*BRCA2a* (At4g00020/10)	CCAATTTCTTTACAGAAGCCTAGTCG	RT-PCR
328	At*BRCA2b* (At5g01630)	GCTCTGAATATCAGTAAACCTGCT	genotyping and RT-PCR
404	At*BRCA2b* (At5g01630)	TGTATCACACGATACAACAGACA	genotyping
799	At*BRCA2b* (At5g01630)	TACAACAGACAAACCACTTGAAGCTTGCT	RT-PCR
177	At*KU80* ((At1g48050)	TGTCTTTTGCTTGTTGTGCAG	genotyping
178	AtKU80 ((At1g48050)	GCAGAAGGTGCAAGGTCAAG	genotyping
456	At*KU80* ((At1g48050)	ATGGCACGAAATCGGGAGGGTTTG	RT-PCR
457	At*KU80* ((At1g48050)	ACGATCAAGAAAGTCTCCAGCTAC	RT-PCR
454	At*KU80* ((At1g48050)	GAAGATTAAGGTGTGGGTTTATAAG	RT-PCR
455	At*KU80* ((At1g48050)	GTAAAACGAATCAGGAGTATCATCTC	RT-PCR
458	At*KU80* ((At1g48050)	CAAGGAGAATCCAAAGTTGAAGAAGG	RT-PCR
459	At*KU80* ((At1g48050)	CGTCTACTATATCACTGTCCGCTG	RT-PCR
510	At*LIGASE6* (At1g66730)	TCATTGCAGAATTGCTAAGGG	genotyping
509	At*LIGASE6* (At1g66730)	GAAGACGCAGACTTCAACCTG	genotyping
637	At*LIGASE6* (At1g66730)	AGAGCACGCTTGTTGGAGGG	RT-PCR
638	At*LIGASE6* (At1g66730)	TAAATTACGGGCCAATGTTCTAACAAG	RT-PCR
686	At*LIGASE6* (At1g66730)	GAGGGTGTTTCTGCTGCAGTAGTTGAGGCTTACAA	RT-PCR
640	At*LIGASE6* (At1g66730)	AAGAGCCAACAGCTGTTCTCCA	RT-PCR
641	At*LIGASE6* (At1g66730)	TTCATGGCTCAAGGTTAAGCGAGAT	RT-PCR
642	At*LIGASE6* (At1g66730)	GTTTGAGCATGAAACATCTCTGCGA	RT-PCR
Act-464	At*ACT2* (At3g18780)	TGAGACCTTTAACTCTCCCG	RT-PCR
Act-465	At*ACT2* (At3g18780)	GATGGCATGAGGAAGAGAGA	RT-PCR
336	At*LIGIV* (At5g57160)	TTGCTGCTGAGGTATTGCAACGTAGAC	RT-PCR
445	At*LIGIV* (At5g57160)	CCATCAAGGATACACTTGTCCACCAAT	RT-PCR
452	At*ERCC1* (At3g05210)	CCCACAGTTCAAGCCAAACGCATC	RT-PCR
453	At*ERCC1* (At3g05210)	ACATTCTGTCATGCTCCAGGCAC	RT-PCR

See figures 1, 4 and 7 for the relative position of the primers.

### RNA isolation and RT-PCR analyses

Total RNA was extracted from leaves or floral buds of 2–3 week-old individual plants with the NucleoSpin® RNA Plant (Macherey-Nagel) according to the manufacturers' specifications. 2 µg of total RNA was used as a template for reverse-transcription with the RT ImProm II™ (Promega) and oligod(T) as a primer. 1/20 of the RT reactions were used as a template for PCR in a total volume of 50 µl. The quality of the RT reaction was controlled by examining actin expression by PCR using primers act-464 and act-465. For At*BRCA2a* and At*BRCA2b* cDNAs, specific primers flanking the T-DNA insertion were designed: 873/798 and 328/799 respectively (see Table 1 and Figure 1). For At*KU80,* specific primers were designed 5′ to the insertion (456/457), flanking the T-DNA insertion (454/455) and 3′ to the T-DNA (458/459) (see Table 1 and Figure 4). For At*LIGIV* and At*ERCC1* cDNAs, specific oligonucleotides were designed flanking their respective T-DNA insertion site: the 336/445 pair for At*LIGIV* and the 452/453 pair for At*ERCC1* (see Table 1 for sequences). For At*LIGASE6*, specific primers were designed 5′ to the insertion (637/638), flanking the T-DNA insertion (686/640) and 3′ to the T-DNA (641/642) (see Table 1 and Figure 7). The PCR was as follows: 94°C for 3 min, followed by 35 cycles at 94°C for 30 s, 50°C or 52°C for 30 s and 72°C for 1 min, except for the *ACTIN* gene (see Table1 for sequences of the ACT2 primers) where the elongation step was performed at 58°C for 30 s. 20 µl of the RT-PCR reaction were then loaded onto a 3% agarose gel (NuSieve) in the presence of ethidium bromide for visualization.

### p*DMC1*:: cDNA At*BRCA2a* construct

The full length cDNA of At*BRCA2a* was previously cloned in pUC18 as described in [39]. It was subsequently subcloned first into pKannibal [55] and then into the *Xho*I–*Spe*I-restricted pPF408 to be set under the p*DMC1* promoter control [39].

### In vitro assays for sensitivity to MMS, gamma-rays and UV

Seeds were surface-sterilized with a solution containing 50% bleach diluted in EtOH. Sterilized seeds were sown on MS 0.5 agar media (Kalys) containing 1% sucrose and supplemented with 0, 50, 60, 70, 80 or 90 ppm of MMS and then set at 4°C in the dark for 48 h to synchronize germination, before being placed vertically in the growth chamber for 14 days to allow the roots to grow along the agar surface. For irradiation experiments, sterilized seeds stored at 4°C in the dark were exposed to 0, 100 or 200 Grays from a ^137^Cs source at a dose rate of approximately 50 gy.min^−1^ (IBL-637 (CIS-BioInternational), Institut Curie, Orsay). After irradiation, they were sown on MS 0.5 agar media and set vertically in a growth chamber. After 11 days, root growth was observed. For UV experiments, sterilized seeds were sown on MS 0.5 agar and after 4 days of vertical growth, the plantlets were exposed to 540 J.m^−2^ of UV-C (254 nm). The plates were then 90 degrees rotated, set in the dark for 3 days to avoid photoreactivation and then exposed 3 days to light to observe the recovery of main root growth of each seedling during two weeks.

### Plant crosses

Since all single mutants used in this study were fertile, double mutants were obtained by crossing two homozygous mutants affected in the gene of interest. Double mutants were identified by PCR of the F2 population obtained by self-fertilisation of F1 plants heterozygous for both genes.

### Transformation of plants with RNAi constructs

The RNAi constructs aimed at silencing both *BRCA2* genes and the control without any insert were previously described in [39]. Plant transformations were carried out by floral dip as described previously [56]. T1 transformants were selected on sand supplemented with Basta® and transferred to soil pots. Approximately one to two weeks after, the selected transformed plants were sprayed with Basta® (4% ammonium glufosinate) for a second control of their resistance.

### DAPI staining and cytology

The flower buds or the siliques were fixed in a solution of absolute ethanol and acetic acid (3/1 v/v) at room temperature. Chromosome spreads were prepared as in [57]. Photographs were captured using a Photometrics CoolSNAP EZ camera driven by Metavue 7.0 r4 software.

Fixed siliques were placed in ethanol 70% during 2 h, and then in a chloralhydrate solution (8 g/3 ml glycerol 66%) during a night in the dark. Images were captured on a Zeiss stereo-microscope Stemi SV1 with a SONY camera driven by Zeiss Axiovision Software.

All images were further processed with Adobe Photoshop CS2.

## Supporting Information

Figure S1 **Observation of meiocytes by DAPI staining in** ***nhej*** **,** ***ssa*** **,** ***nhej ssa*** **and** ***lig6*** **mutant plants transformed with the RNAi/0 control construct.** Normal meiotic progression in plants transformed with p*DMC1*::RNAi/0 in *nhej* mutant plants, *ku80* (A–B) and *lig4* (C–D), in the *ssa* mutant *ercc1* (E–F), in double *nhej ssa* mutants *ku80 ercc1* (G–H) and *lig4 ercc1* (I–J), and in *lig6* mutant plants (K–L). Bivalents were correctly associated during the first meoitic phase (diakinesis (A–E–G–K) and metaphaseI (C, I). Segregation of homologous chromosomes and during the second division, sister chromatid separation occurred normally without chromosomal bridges or fragmentation (metaphase II or early anaphase II (L), anaphase II (B–D–H–J) plants, and telophase II (F)). Bar 10 µm.
(TIF)
