# Supplementary figures and images for: Characterization of Brca2-Deficient Plants Excludes the Role of NHEJ and SSA in the Meiotic Chromosomal Defect Phenotype
Source: PLoS One. 2011 Oct 21;6(10):e26696. doi: 10.1371/journal.pone.0026696 (PMC3198793; doi:10.1371/journal.pone.0026696)

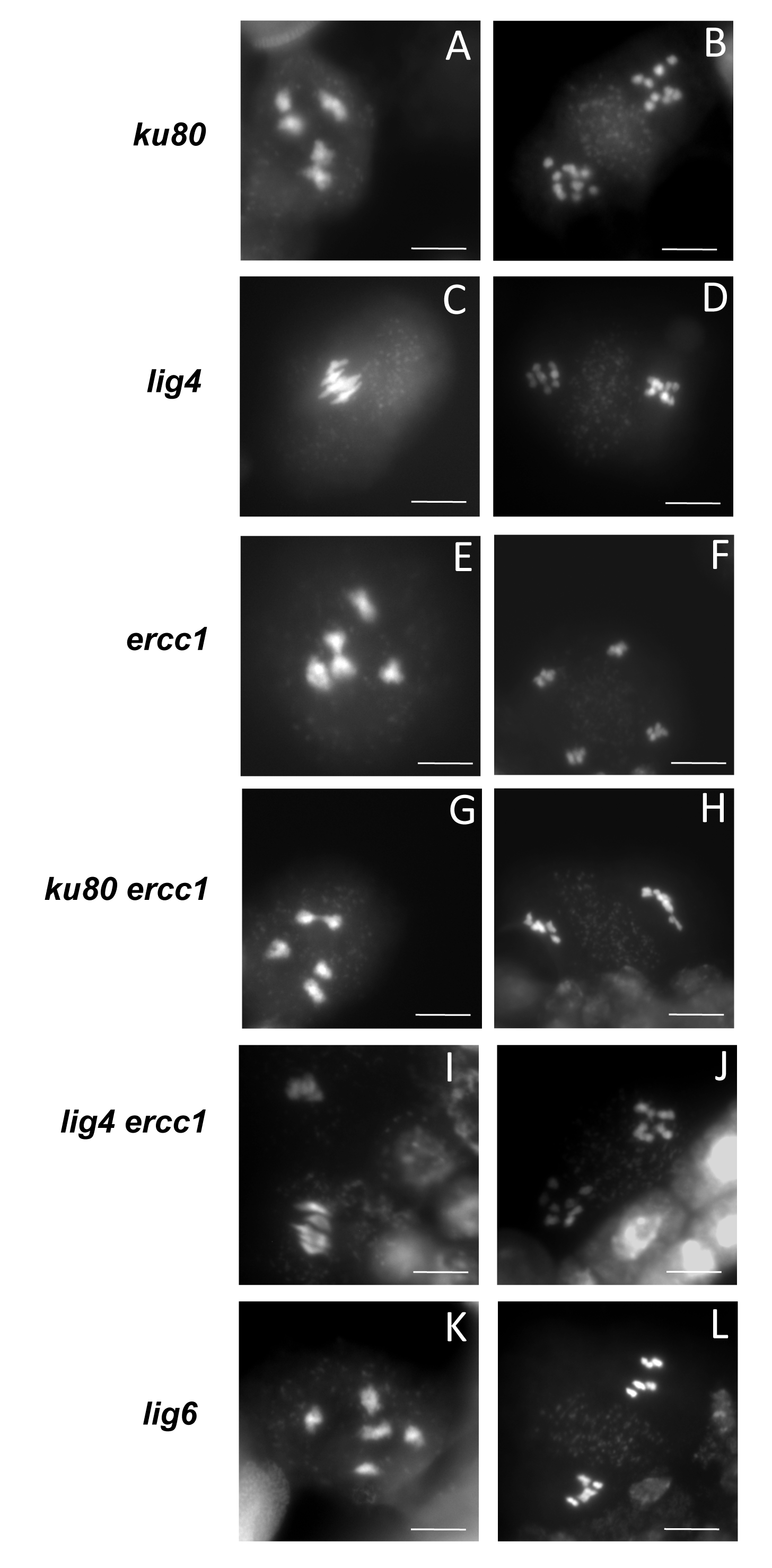

Supplement: Figure S1 — Observation of meiocytes by DAPI staining in nhej , ssa , nhej ssa and lig6 mutant plants transformed with the RNAi/0 control construct. Normal meiotic progression in plants transformed with pDMC1::RNAi/0 in nhej mutant plants, ku80 (A–B) and lig4 (C–D), in the ssa mutant ercc1 (E–F), in double nhej ssa mutants ku80 ercc1 (G–H) and lig4 ercc1 (I–J), and in lig6 mutant plants (K–L). Bivalents were correctly associated during the first meoitic phase (diakinesis (A–E–G–K) and metaphaseI (C, I). Segregation of homologous chromosomes and during the second division, sister chromatid separation occurred normally without chromosomal bridges or fragmentation (metaphase II or early anaphase II (L), anaphase II (B–D–H–J) plants, and telophase II (F)). Bar 10 µm. (TIF) [file pone.0026696.s001.tif]
